# Pseudoangiosarcoma and cutaneous collagenous vasculopathy in a patient on a Bruton’s tyrosine kinase inhibitor

**DOI:** 10.1016/j.jdcr.2024.06.011

**Published:** 2024-07-01

**Authors:** Alison H. Kucharik, Nina B. Curkovic, Julio C. Chavez, Kenneth Y. Tsai, Andrew S. Brohl, James M. Grichnik

**Affiliations:** a Department of Dermatology and Cutaneous Surgery, University of South Florida Morsani College of Medicine, Tampa, Florida; b Department of Medicine, Vanderbilt University School of Medicine, Nashville, Tennessee; c Department of Malignant Hematology, H. Lee Moffitt Cancer Center and Research Institute, Tampa, Florida; d Department of Pathology, Division of Dermatopathology, Moffitt Cancer Center, Tampa, Florida; e Department of Cutaneous Oncology, Moffitt Cancer Center, Tampa, Florida

**Keywords:** angiosarcoma, Bruton’s tyrosine kinase inhibitor, BTKi, CCV, cutaneous collagenous vasculopathy

## Introduction

Adverse events of Bruton’s tyrosine kinase inhibitors (BTKis) used in the treatment of chronic lymphocytic leukemia (CLL) and other B-cell malignancies commonly include ecchymoses, purpura, and petechiae. Although second-generation BTKi are more selective than the first-generation BTKi, low-grade bleeding adverse events occur at similar rates in approximately one-third of patients.^1^ Several case reports describe an extensive ecchymotic patch on sun-exposed extremities in patients treated with BTKi, suggesting the role of repeated microtrauma and UV exposure. We present a case of a similar appearing ecchymotic patch in which targeted biopsies initially favored angiosarcoma, although later was reclassified as cutaneous collagenous vasculopathy (CCV) as the lesion improved off therapy. This suggests that BTKi may drive focal endothelial proliferations, which may be the underlying cause of the associated vascular leakage. The molecular cause of this process is unknown but could be related to nonreceptor site binding/activation of G protein subunit alpha q (GNAQ) or other impacts on epidermal growth factor receptor (EGFR), Src, and tyrosine kinase expressed in hepatocellular carcinoma (TEC) family kinases.

## Case report

**Fig 1:**
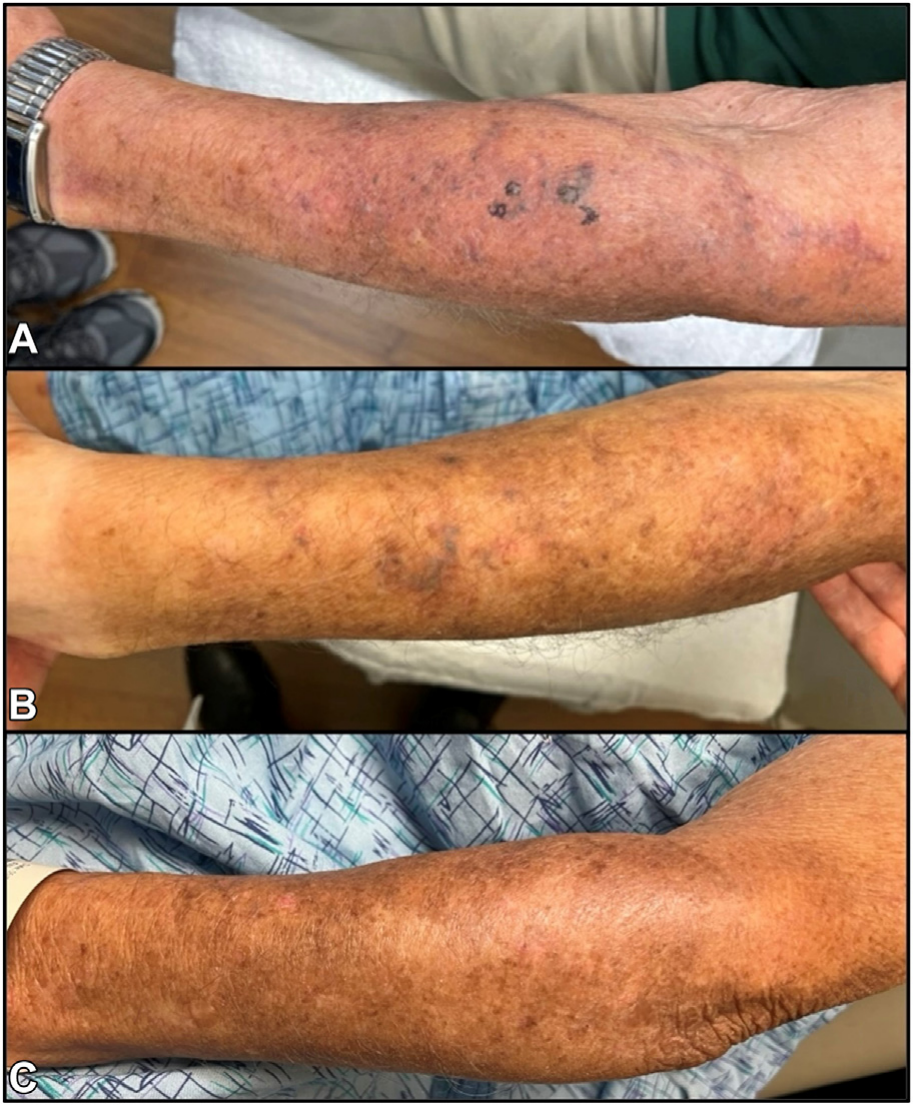
Clinical course of our patient on Bruton’s tyrosine kinase inhibitors (BTKis). **A,** Initial presentation to oncology and dermatology: dorsal aspect of the left forearm with an extensive red-to-purple ecchymotic patch with subtle, blue, multifocal dermal papules. **B,** Two days after discontinuation of BTKi with the presence of midforearm blue/purple papules. **C,** Four months after discontinuation of BTKi: disappearance of blue/purple papules and further lightening of ecchymoses to brown macules and patches suggestive of hemosiderin deposition in postinflammatory hyperpigmentation.

An 87-year-old man with a history of CLL treated with acalabrutinib presented with an ecchymotic patch on his left forearm (Fig 1). At the recommendation of his oncologist, he discontinued acalabrutinib and the patch began to fade. However, his CLL progressed, and he restarted acalabrutinib, leading to the reappearance and darkening of the ecchymotic patch.

**Fig 2:**
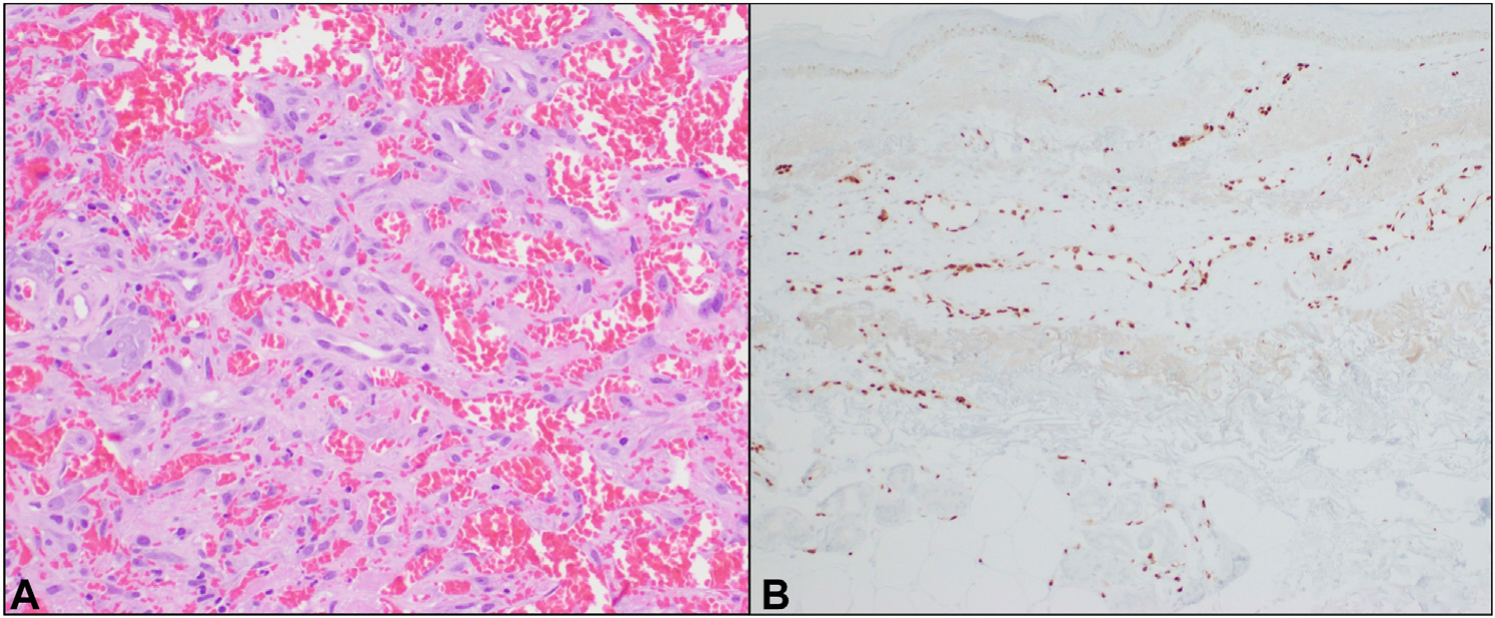
Initial punch biopsies showed **(A)** papillary dermal anastomosing ectatic vascular spaces lined by large atypical cells and **(B)** ERG stain highlighting large atypical endothelial cells.

This prompted his presentation to the dermatology clinic, where he exhibited an extensive red-to-purple ecchymotic patch with subtle, blue, multifocal dermal papules (Fig 1). Pertinent laboratory studies within 1 month of the presentation included a platelet count of 250 (K/μL), an international normalized ratio of 0.9, and an activated partial thromboplastin time of 34.8 seconds. Two punch biopsies of these papules revealed papillary dermal anastomosing ectatic vascular spaces lined by atypical cells. Immunohistochemical staining was positive for ERG and CD31, suggestive of angiosarcoma (Fig 2).

Notably, he had no history of radiation or lymphedema, and he had no similar lesions on the head or neck area. A positron emission tomography scan revealed a lack of avidity of the left forearm. Given this atypical presentation for cutaneous angiosarcoma and several similar reports of patients with ecchymotic patches that disappeared after the discontinuation of BTKis (Table I),^2^, ^3^, ^4^, ^5^, ^6^ his medical teams were hesitant to initiate aggressive angiosarcoma treatment. Thus, the decision was made to switch to a different BTKi (pirtobrutinib), during which the ecchymotic lesions persisted. He then discontinued BTKis altogether, and the patches and papules began to fade (Fig 1).

**Table I:** Case reports and case series documenting petechial, purpuric, and/or ecchymotic cutaneous manifestations in patients treated with BTKi

BTKi	Disease	Age, y/sex	Location	Pathology	Outcome	Reference
Acalabrutinib, pirtobrutinib	CLL	87 M	Dorsal aspect of the left forearm	First biopsy: papillary dermal anastomosing ectatic vascular spaces lined by atypical cells. IHC positive for ERG and CD31, indicative of angiosarcoma. Second biopsy: dermal telangiectasia and dilated vascular structures with deposits in the vessel walls. PAS stain highlighted hyaline membrane material around blood vessels consistent with cutaneous collagenous vasculopathy.	Improvement 9 d after discontinuation of BTKi	
Acalabrutinib	CLL	68 F	Dorsal aspect of the left forearm	Superficial vascular ectasia and chronic thrombotic changes with partial occlusion of the superficial and middermal vessels by proteinaceous debris. Dermis with perivascular lymphohistiocytic infiltrate and extravasated RBCs.	Resolution 6 wk after discontinuation of BTKi	Guenther et al^2^
Acalabrutinib	SLL	80 M	Bilateral dorsal and ventral aspect of the upper extremities	Mild interface reaction with superficial and deep lymphocytic infiltrate and presence of eosinophiles. Also evidence of noninflammatory purpura with extravasation of RBCs.	Improvement 4 mo after discontinuation of BTKi	Truong et al^3^
Zanubrutinib	DLBCL	71 F	Right side of the upper extremity and chest wall	Biopsy not performed.	BTKi discontinued, but patient experienced skin necrosis thought to be secondary to DLBCL progression	Wang et al^4^
Ibrutinib	CLL	81 M	Bilateral dorsal aspect of the forearms/hands	Biopsy not performed.	Resolution after discontinuation of BTKi	Jani et al^5^
Acalabrutinib	CLL (2), MCL (1)	70–78 M and F	Left arm for one patient, unspecified for others	One patient’s biopsy: vascular ectasia with nonocclusive thrombotic vasculopathy.	Resolution after discontinuation of BTKi or with switch to different BTKi	Schmidt et al^6^

*BTKi*, Bruton’s tyrosine kinase inhibitor; *CLL*, chronic lymphocytic leukemia; *DLBCL*, diffuse large B cell lymphoma; *F*, female; *IHC*, immunohistochemical staining; *M*, male; *MCL*, mantle cell lymphoma; *RBC*, red blood cell; *SLL*, small lymphocytic leukemia.

**Fig 3:**
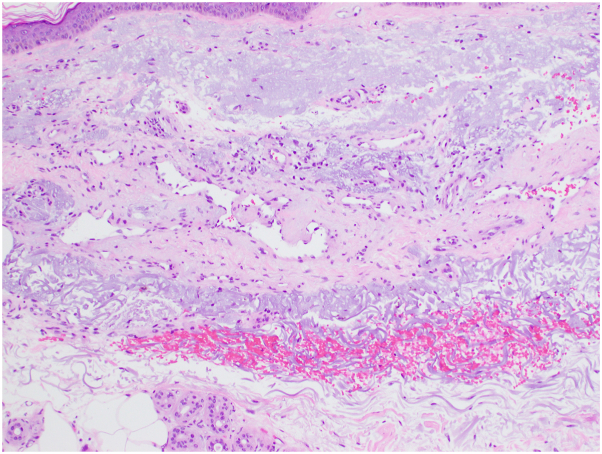
Dermal telangiectasia and dilated vascular structures with deposits in the vessel walls.

Nine days after discontinuation of BTKi, a rebiopsy of an ecchymotic patch directly adjacent to the previously biopsied and resolved papules revealed dermal telangiectasia and dilated vascular structures with deposits in the vessel walls. PAS stain highlighted hyaline membrane material around blood vessels. ERG stain highlighted endothelial cells that showed a low-absent proliferative rate by Ki67 stain. D2-40 highlighted lymphatic channels. HHV8 stain was negative (Fig 3). These findings, along with the clinical course, were suggestive of CCV secondary to BTKi therapy.

## Discussion

Bleeding is a well-known complication of BTKi treatment. In this case, a focused biopsy of the dark purple papules within the lesion revealed changes concerning angiosarcoma. The development of primary angiosarcoma has been reported in a clinical trial for acalabrutinib (*n* = 1).^7^ However, the impact of BTKi on the risk of additional primary or secondary malignancies has not been elucidated. Unanticipated development of secondary malignancies and hyperproliferative skin conditions during treatment with other kinase inhibitors, notably BRAF inhibitors, has been established to be the result of paradoxical activation of other signaling pathways.^8^ The authors posit that this case may illustrate a similar paradoxical activation pathway with BTKi, for example, the GNAQ, EGFR/SRC/TEC-family kinases, or proapoptotic endothelial signaling pathways.

It is well known that GNAQ-activating mutations play a role in various vascular anomalies and proliferations.^9^ Ma et al^10^ demonstrated that a nonreceptor site of BTK binds GNAQ. It is possible that BTKi inhibition results in activation of GNAQ, driving focal endothelial proliferation and vascular fragility, leading to the bleeding events seen with BTKi. Furthermore, the off-target inhibition of EGFR, Src, and TEC-family kinase inhibitors by BTKi may also drive this proliferative process in endothelial cells. Further, it has also been suggested that these pathways may directly be implicated in bleeding events, through modified signaling via platelet membrane receptors such as glycoprotein VI and Von Willebrand factor.^1^ Lastly, Liu et al^11^ recently demonstrated that BTKi can upregulate proapoptotic gene expression and endothelial dysfunction, which may contribute to endothelial damage.

CCV was first described in 2000, and there have only been approximately 60 reported cases, some of which have been related to medications. CCV is a rare form of cutaneous microangiopathy that typically presents as localized telangiectasias that may become generalized. Histopathology demonstrates ectasia of superficial vessels with thickened walls due to perivascular collagen deposition.^12^ To the authors’ knowledge, CCV has not been reported in association with angiosarcoma, or BTKi.

The pathogenesis of CCV has not been elucidated. It has been postulated that the collagen deposition and intravascular occlusion by fibrin, both of which have been reported in patients on BTKi (Table I), are part of the natural reparative response to vascular injury.^12^^,^^13^ The vascular injury seen in BTKi-associated CCV may be in response to BTKi effects on GNAQ, EGFR/Src/TEC-family kinases, and endothelial apoptotic signaling pathways, as discussed above.

The authors hypothesize that the angiosarcoma-like pathology and CCV pathology findings found in this case may be 2 entities on the same developmental spectrum of aberrant angiogenesis and vascular dysfunction induced by BTKi. We suspect the focal purple papules represent endothelial proliferations that result in vascular leakage and the surrounding ecchymosis. Although we suspect these endothelial proliferations will resolve off therapy, it is not clear if that will be true in all cases. The molecular pathways driving this process remain to be elucidated. Further research is warranted.

## Conflicts of interest

None disclosed.
